# BUILDING NETWORKS OF PROFESSIONALS TO SUPPORT KINSHIP CARE FAMILIES

**DOI:** 10.1093/geroni/igz038.1043

**Published:** 2019-11-08

**Authors:** Andrea B Smith

**Affiliations:** Western Michigan University, Kalamazoo, Michigan, United States

## Abstract

Numbers of American children being raised by relatives continue to rise. Over 7.8 million children, representing 10.5% of American children under age 18, lived in a relative-headed home. Of these, 2.5 million have no parent present in their home (Generations United, 2016). Kinship families’ needs are typically complex, needing prompt and sensitive responses. Varied professionals often serve kinship families but the majority report receiving little information or training related to kinship families. (Smith 2017 This pilot project surveyed professionals (n = 63) representing varied disciplines in a pre/post-test format to determine change in knowledge and strategies for working with kinship care family members. All respondents were enrolled in graduate courses specifically focused on kinship families. Respondents represented diverse fields including family therapists (n = 9), family service workers (n = 23), teachers (n = 16), school administrators (n = 4), child care providers (n = 2) and health care professionals (n = 7 ). Respondents completed a 17 question pre/post survey. Results demonstrated the majority (n = 59; 93%) had experience with kinship families but most (n = 47; 74%) had received little/no targeted professional training. Post-test results strongly indicated that completing any amount of kinship-related coursework positively impacted professionals’ knowledge, confidence, and readiness to utilize learned strategies in their work with kinship families. Additionally, post-test results of students completing the entire series of classes (9 credits) revealed significantly greater changes, demonstrating the importance of providing comprehensive information to enhance professional practices for working with kinship families.

